# A Laser-Based Measuring System for Online Quality Control of Car Engine Block

**DOI:** 10.3390/s16111877

**Published:** 2016-11-08

**Authors:** Xing-Qiang Li, Zhong Wang, Lu-Hua Fu

**Affiliations:** State Key Laboratory of Precision Measuring Technology and Instruments, Tianjin University, Tianjin 300072, China; lxq.792751045@163.com (X.-Q.L.); wangzhong@tju.edu.cn (Z.W.)

**Keywords:** optical inspection, inner dimension, laser triangulation sensor

## Abstract

For online quality control of car engine production, pneumatic measurement instrument plays an unshakeable role in measuring diameters inside engine block because of its portability and high-accuracy. To the limitation of its measuring principle, however, the working space between the pneumatic device and measured surface is too small to require manual operation. This lowers the measuring efficiency and becomes an obstacle to perform automatic measurement. In this article, a high-speed, automatic measuring system is proposed to take the place of pneumatic devices by using a laser-based measuring unit. The measuring unit is considered as a set of several measuring modules, where each of them acts like a single bore gauge and is made of four laser triangulation sensors (LTSs), which are installed on different positions and in opposite directions. The spatial relationship among these LTSs was calibrated before measurements. Sampling points from measured shaft holes can be collected by the measuring unit. A unified mathematical model was established for both calibration and measurement. Based on the established model, the relative pose between the measuring unit and measured workpiece does not impact the measuring accuracy. This frees the measuring unit from accurate positioning or adjustment, and makes it possible to realize fast and automatic measurement. The proposed system and method were finally validated by experiments.

## 1. Introduction

On car engine production lines, the dimensional measurement of shaft holes inside engine block is critical and difficult, and has a great impact on ultimate product quality [1]. The tactile caption of the coordinate measuring machine (CMM) is a conventional choice for the parameters of shaft holes [2]. CMM has the advantage of its precision and flexibility with respect to various measured objects. However, CMM requires significant resources in operating time, environment and skillful operators, which leads researches into other directions [3,4]. Pneumatic micrometer is another alternative for assessment of machined holes on the shop floor [5]. Its measurement principle is based on the variation of pressure, which is proportional to the distance between the bore gauge nozzles and the measured object. Pneumatic micrometer can achieve rather high accuracy to 0.5 μm, whereas its working range is usually as small as dozens of micron [6,7]. Consequently, this type of devices usually requires manual operation, and it is difficult to integrate pneumatic micrometer into automatic production line.

An ideal measuring method and system in production engineering needs to be automatic, fast and accurate. To meet these requirements, however, there are two difficulties in measuring shaft holes inside engine block. Firstly, shaft holes inside engine block are small and deep, and generally distributed on multi-layers. Secondly, to fulfill the demand for production efficiency, the dimension inspection needs to be done on site and in minutes. For example, there are five-layer shaft holes to be measured inside the 4 H engine block by Dongfeng Motor Co., Ltd. (Wuhan, China). The diameter of the shaft holes is as small as 92 mm. The depth of these shaft holes is approximately 550 mm. In the production line of 4 H engine blocks, the accuracy requirement of inner diameters is 5 μm and the objective parameters should be measured within 4 min. Researches on inner dimension inspection have been studied in some literature. A slender tactile probe was developed for contour and roughness measurements within deep and narrow holes [8]. Owing to its high positioning accuracy and small size, this type of probes is well suited for micro holes [8,9,10]. Nevertheless, by this means, feature points should be measured in sequence, and this way lowers the measuring efficiency. In [11,12], parameters inside machined holes were measured by means of optical scanning. Optical scanning method is simple and fast, but is easily disturbed by angle errors caused by angle encoder and radial run-out caused by revolving spindle [13,14]. Zavyalov completed 3D hole inspection using lens with high field curvature [15]. Without rotary scanning or other support devices, using such lens seems a promising solution to simplify the system set-up. It cannot be ignored, however, the accuracy of this approach (0.1 mm level) is not high enough for accurate measurement. For this purpose, this type of methods is commonly used for defect detection or shape measurement, such as [16,17,18]. An automated inner dimensional measurement system was proposed based on a laser triangulation sensor (LTS) for long-stepped pipes [19]. However, the special purpose system cannot be applied to shaft holes inside engine block.

In this article, we developed a high-speed automatic measuring system for shaft holes inside engine block. Compared with such probes in [8,9,10], the proposed system can measure multi-parameters simultaneously and its measuring efficiency can meet the requirements of lean production. Different from [11,12], the measuring unit utilizes the layout of pneumatic micrometer, and this arrangement cannot be affected by dynamic factors. To overcome the short range of pneumatic micrometer, LTSs [20,21,22], which have the advantage of high accuracy and wide measuring range, are employed in our system to acquire raw data. The measuring principle of LTS is introduced in Section 2. The configuration of proposed system is then presented in Section 3. Section 4 describes in detail the mathematical measurement model and data processing algorithm. Experiments are performed in Section 5 to validate proposed measuring method and system. The conclusions are finally given in Section 6.

## 2. Measuring Principle of Laser Triangulation Sensor

**Figure 1:**
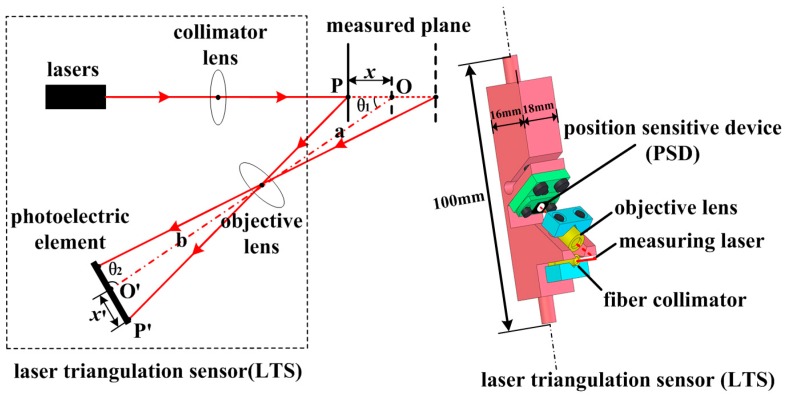
Principle of laser triangulation sensor.

Laser triangulation sensors (LTSs) have the advantage of wide measuring range and high accuracy, and are commonly used in dimensional metrology (in addition to reverse engineering applications). A LTS is mainly composed of two sub-systems: one is the laser emitting system, which employs a semiconductor laser; and the other is the laser receiving system, which employs a photoelectric element. The principle of LTS is shown in Figure 1. The incident laser goes through collimator lens and points at the measured object. The reflected light spreads through objective lens and focuses on the photoelectric element. When the laser spot moves from point *P* to point *O*, the corresponding image spot follows from point *P′* to point *O′*. The displacement relationship between the laser point and corresponding image spot is determined by

(1) $$x=\frac{a\cdot x^{\prime }\cdot \text{sin}\;\theta_{2}}{b\cdot \text{sin}\;\theta_{1}-x^{\prime }\cdot \text{sin}(\theta_{1}+\theta_{2})}$$

where *x* is denoted as the displacement of the laser spot; *x′* is denoted as the displacement of the image spot; *a* is the distance between front principal plane of objective lens and the intersecting point of incident laser with optical axis of objective lens; *b* is the distance between rear principal plane of objective lens and the center of image plane; *θ*_1_ is the angle between incident laser and optical axis of objective lens; and *θ*_2_ is the angle between image plane and optical axis of objective lens.

The LTS works only if the incident laser is approximately perpendicular to the measured object. As a result, the defocus phenomenon exists among all the image spots but the sole focus one. This would introduce the light interference when estimating the image spot’s position and finally impact the measuring accuracy. To improve this, the optical system needs to follow the Scheimpflug rule [23]. Then, Equation (2) should be satisfied:

(2) $$\text{tan}\;\theta_{1}=\frac{b}{a}\cdot \text{tan}\;\theta_{2}$$

By inserting Equation (2) into Equation (1), the displacement relationship can be finally expressed as

(3) $$x=\frac{a\cdot \text{sin}\;\theta_{2}}{b\cdot \text{sin}\;\theta_{1}}\cdot x^{\prime }\left(a\gg \frac{\text{sin}(\theta_{1}+\theta_{2})}{\text{sin}\;\theta_{2}},\frac{\theta_{1}}{2}+\theta_{2}>\frac{\pi }{2}\right)$$

Commercial LTSs have been widely used for dimensional measurement. However, their size or accuracy specifications cannot meet the requirements of the proposed measuring system [24]. Hence, a LTS is developed in this article to measure the shaft holes inside engine block. To place developed LTS inside the narrow measured hole, the structure of the developed LTS should be compact enough. Consequently, position sensitive device (PSD) is chosen as the photoelectric element because of its small size and simple processing circuit. Measuring accuracy is another key parameter needs to be deeply concerned. As mentioned above, the Scheimpflug rule could weaken the defocus phenomenon, which benefits the measuring accuracy. However, the Scheimpflug rule cannot solve this problem thoroughly. To improve this, only part of the PSD around the focus is selected as the effective measuring range. This would further decrease the influence of defocus phenomenon and thus improve the accuracy at the cost of decreasing the measuring range. In addition, fiber-delivered laser is employed instead of internal laser source to further decrease the sensor’s size and reduce thermal accumulation in LTS generated during measurement process, which would cause temperature drift and thus affect the measuring accuracy. In the following section, the developed LTSs will be integrated into the measuring system to acquire the data samples.

## 3. System Configuration

**Figure 2:**
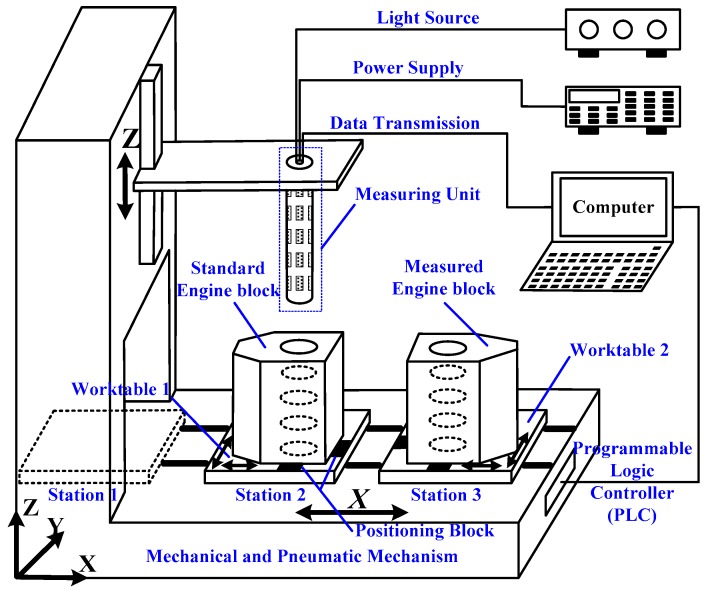
The measuring system.

The high-speed, automatic measuring system for shaft holes inside engine block, as shown in Figure 2, mainly consists of the mechanical and pneumatic mechanism, a measuring unit, data processing system, and standard and measured workpieces. This measuring system is specially designed for the 4 H engine block by Dongfeng Motor Co., Ltd., in which five-layer shaft holes needs to be measured. Nevertheless, the system design and proposed measuring method can also be applied to other models of engine blocks by simply replacing this measuring unit with another suitable one.

The mechanical and pneumatic mechanism is used just as delivery and transportation, and its positioning accuracy does not impact the final results. Worktables that move along *x*-axis are driven by double-acting cylinders. Worktable 1 travels between Stations 1 and 2, whereas Worktable 2 travels between Stations 2 and 3. Positioning blocks and stepper motors on worktables are employed to adjust the position of engine blocks, to enable the measuring unit to go through measured shaft holes. Driven by servo motors, the measuring unit can move along *z*-axis.

**Figure 3:**
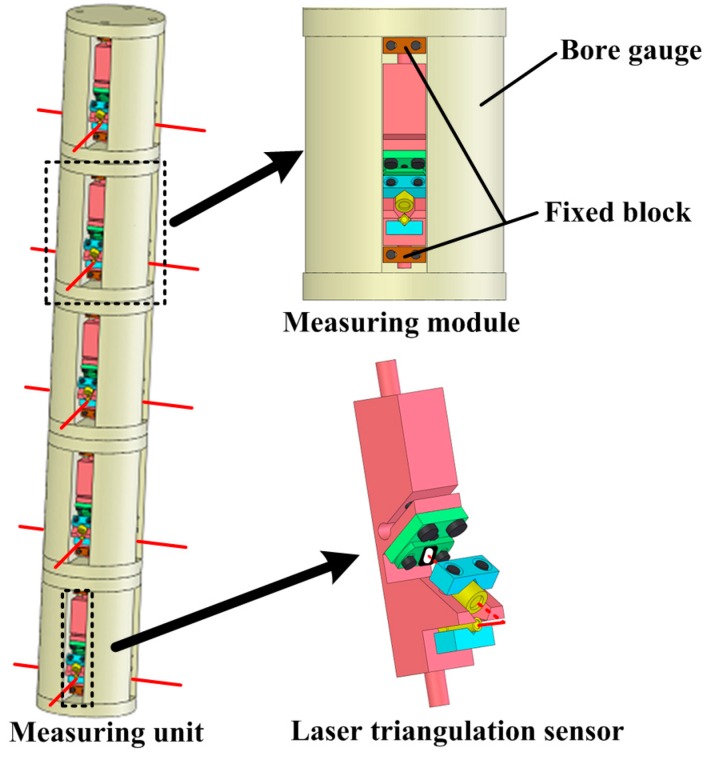
The measuring unit.

The measuring unit, as shown in Figure 3, comprises several measuring modules, which can be taken apart from each other. The number of the measuring modules is determined by the number of measured shaft holes. For example, five measuring modules are involved in our system to make up of the measuring unit, as the 4 H engine block has five-layer shaft holes to be measured. The structure of the measuring module includes a bore gauge and four LTSs. The size of the bore gauge is designed according to measured shaft holes. Four LTSs are arranged in the internal circumference of the bore gauge, at equal intervals, one LTS per quadrant. It is worth mentioning that the measuring unit can be customized to adjust different types of engine blocks by changing the number of the measuring modules and the size of the bore gauge. The measuring data acquired by the measuring unit are transmitted to the data processing system through RS-485 bus. The measuring results are finally computed through data processing system.

**Figure 4:**
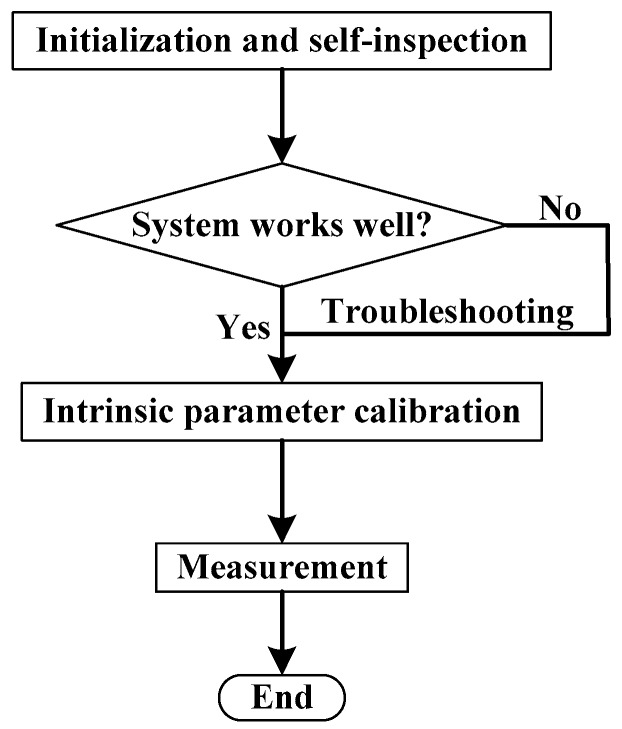
The measurement procedure.

The measurement procedure is shown in Figure 4. “Self-inspection” is to check for a clear data communication and a full control of mechanical and pneumatic mechanism. After that, the relative pose between the LTSs are calibrated by the standard engine block, whose objective parameters have been measured by a high-accuracy CMM. Measurements are performed afterwards. In what follows, the mathematical model for calibration and measurement are described in detail.

## 4. Mathematical Model and Data Processing Algorithm

### 4.1. Mathematical Model

**Figure 5:**
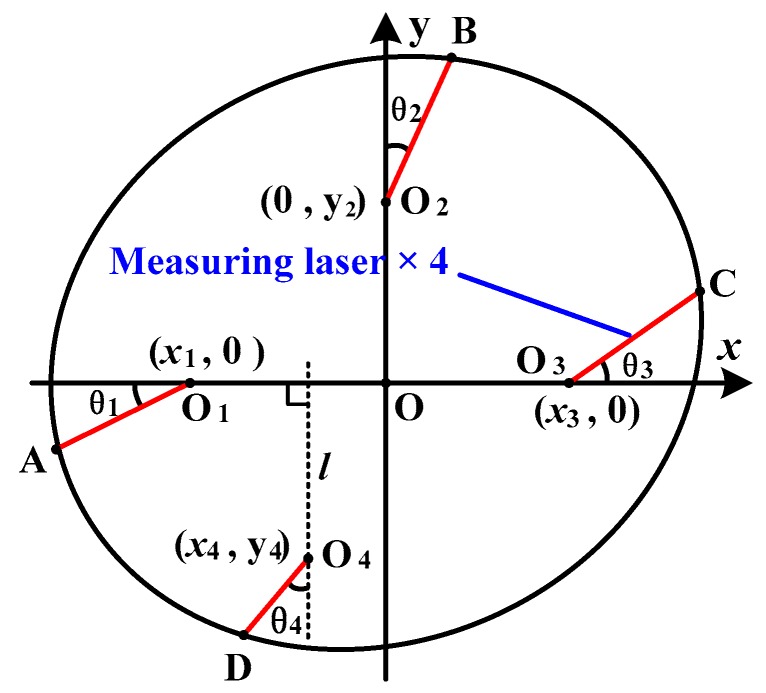
Single-module measurement model.

In this article, the measuring unit consists of twenty LTSs. The complicated relative pose among such numbers of LTSs is difficult to acquire. However, it is not necessary to obtain the relationship among all the LTSs. As shown in Figure 3, the four LTSs arranged on one measuring module are free from the LTSs arranged on other ones. Therefore, the calibration work can be done module by module. A single-module measurement model is established, as shown in Figure 5. The other measuring modules can be calibrated in the same way.

As shown in Figure 5, the shaft axis of the bore gauge may not be parallel to the shaft axis of measured holes. Therefore, in general settings, the measured cross section is considered as an ellipse. *O*_1_, *O*_2_, *O*_3_, and *O*_4_ are the four sensors’ starting points of their measuring ranges, and the measured points are signed as *A*, *B*, *C*, and *D*, respectively. To formulate the relationship between measuring lasers and measured points, Cartesian coordinate system *x*-*o*-*y* is introduced, where *O*_1_, *O*_3_ are, respectively, on the negative and positive *x*-axis, and *O*_2_ is on the positive *y*-axis. The coordinates of *O*_1_, *O*_2_, *O*_3_, and *O*_4_ in coordinate system *x*-*o*-*y* are respectively defined as (*x*_1_, 0), (0, *y*_2_), (*x*_3_, 0), and (*x*_4_, *y*_4_), respectively. The coordinates of *A*, *B*, *C*, and *D* in coordinate system *x*-*o*-*y* are, respectively, defined as (*x*_A_, *y*_A_), (*x*_B_, *y*_B_), (*x*_C_, *y*_C_), and (*x*_D_, *y*_D_). |*AO*_1_|, |*BO*_2_|, |*CO*_3_|, and |*DO*_4_| are the displacements of the four sensors, whose values are, respectively, defined as *l*_1_, *l*_2_, *l*_3_, and *l*_4_. Then, the relationship between measuring lasers and measured points can be expressed as:

(4) $$\begin{array}{l} \{\begin{array}{l} x_{A}=x_{1}-l_{1}\cdot \text{cos}\;\theta_{1}\\ y_{A}=l_{1}\cdot \text{sin}\;\theta_{1} \end{array}\\ \{\begin{array}{l} x_{B}=l_{2}\cdot \text{sin}\;\theta_{2}\\ y_{B}=y_{2}+l_{2}\cdot \text{cos}\;\theta_{2} \end{array}\\ \{\begin{array}{l} x_{C}=x_{3}+l_{3}\cdot \text{cos}\;\theta_{3}\\ y_{C}=-l_{3}\cdot \text{sin}\;\theta_{3} \end{array}\\ \{\begin{array}{l} x_{D}=x_{4}-l_{4}\cdot \text{sin}\;\theta_{4}\\ y_{D}=y_{4}-l_{4}\cdot \text{cos}\;\theta_{4} \end{array} \end{array}$$

where *θ*_1_ is the angle between *O*_1_*A* and the negative *x*-axis, *θ*_2_ is the angle between *O*_2_*B* and the positive *y*-axis, *θ*_3_ is the angle between *O*_3_*C* and the positive *x*-axis, and *θ*_4_ is the angle between *O*_4_*D* and line *l*.

**Figure 6:**
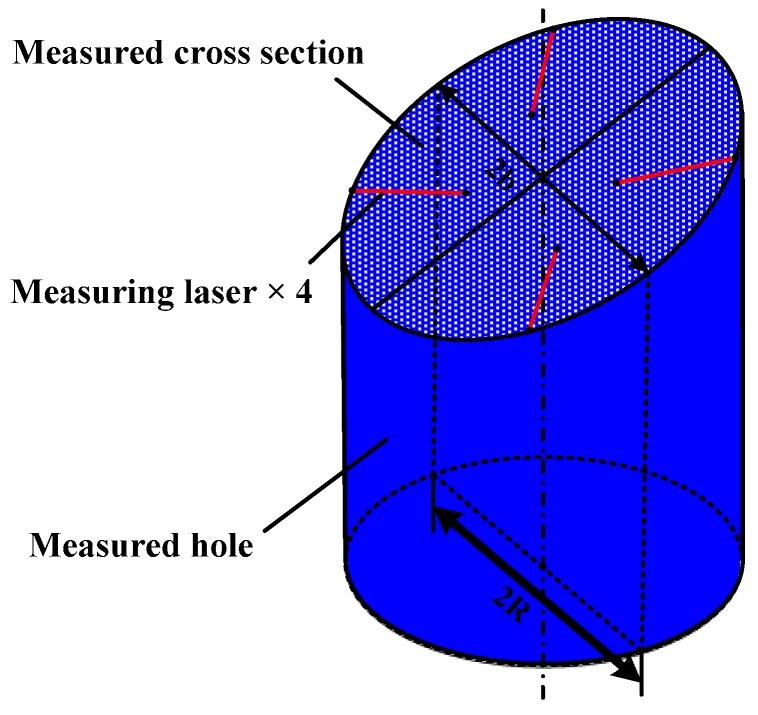
3D measurement model.

Next, we should obtain the relationship between the measured points and measured diameter. For the sake of convenient calculation, the measured cross section whose shape is an ellipse is expressed as:

(5) $$\frac{{\left(x^{E}-m\right)}^{2}}{a^{2}}+\frac{{\left(y^{E}-n\right)}^{2}}{b^{2}}=1$$

where (*m*, *n*) represents the geometrical center of the ellipse in coordinate system *o-xyz*, *a* and *b*, respectively, denote the semi-major axis and the semi-minor axis. The transformation matrix between coordinate system *o*-*xyz* and coordinate system *o-x^E^y^E^z^E^* is:

(6) $$\left[\begin{array}{c} x^{E}\\ y^{E}\\ z^{E} \end{array}\right]=\left[\begin{array}{c} x\\ y\\ z \end{array}\right]\cdot \left[\begin{array}{ccc} \text{cos}\;\gamma & \text{sin}\;\gamma & 0\\ -\text{sin}\;\gamma & \text{cos}\;\gamma & 0\\ 0 & 0 & 1 \end{array}\right]$$

where *γ* represents the rotation angle between coordinate system *o*-*xyz* and coordinate system *o-x^E^y^E^z^E^*, (*p*, *q*) is the translation vector. From Figure 6, we can come to a significant conclusion: regardless of the relative pose between the measuring module and measured shaft hole, the diameter of measured shaft hole is always equal to the ellipse’s semi-minor axis *b* [25]. Consequently, Equation (5) can be expressed in another form:

(7) $$\frac{{\left(x^{E}-m\right)}^{2}}{a^{2}}+\frac{{\left(y^{E}-n\right)}^{2}}{R^{2}}=1$$

where *R* is the value of measured diameter. By inserting the coordinates of the measured points into Equation (7), we can obtain the relationship between the measured points and measured diameter:

(8) $$\{\begin{array}{l} \frac{{\left(x_{A}^{E}-m\right)}^{2}}{a^{2}}+\frac{{\left(y_{A}^{E}-n\right)}^{2}}{R^{2}}=1\\ \frac{{\left(x_{B}^{E}-m\right)}^{2}}{a^{2}}+\frac{{\left(y_{B}^{E}-n\right)}^{2}}{R^{2}}=1\\ \frac{{\left(x_{C}^{E}-m\right)}^{2}}{a^{2}}+\frac{{\left(y_{C}^{E}-n\right)}^{2}}{R^{2}}=1\\ \frac{{\left(x_{D}^{E}-m\right)}^{2}}{a^{2}}+\frac{{\left(y_{D}^{E}-n\right)}^{2}}{R^{2}}=1 \end{array}$$

where ($x_{A}^{E}$,$y_{A}^{E}$), ($x_{B}^{E}$,$y_{B}^{E}$), ($x_{C}^{E}$,$y_{C}^{E}$) and ($x_{D}^{E}$,$y_{D}^{E}$) are, respectively, the coordinates of *A*, *B*, *C* and *D* in coordinate system *o-x^E^y^E^z^E^*. By solving Equation (8), *m*, *n* and *a* can be expressed as follows:

(9) $$\{\begin{array}{l} m=\frac{1}{2}\cdot \frac{A}{B}\cdot \frac{1}{C}\cdot \left[\left(y_{A}^{E}-y_{B}^{E}\right)\cdot \left(y_{B}^{E}-y_{C}^{E}\right)\cdot \left(y_{A}^{E}-y_{C}^{E}\right)+\left(y_{B}^{E}-y_{C}^{E}\right)\cdot {\left(x_{A}^{E}\right)}^{2}+\left(y_{C}^{E}-y_{A}^{E}\right)\cdot {\left(x_{B}^{E}\right)}^{2}+\left(y_{A}^{E}-y_{B}^{E}\right)\cdot {\left(x_{C}^{E}\right)}^{2}\right]\\ n=-\frac{1}{2}\cdot \frac{B}{A}\cdot \frac{1}{C}\cdot \left[\left(x_{A}^{E}-x_{B}^{E}\right)\cdot \left(x_{B}^{E}-x_{C}^{E}\right)\cdot \left(x_{A}^{E}-x_{C}^{E}\right)+\left(x_{B}^{E}-x_{C}^{E}\right)\cdot {\left(y_{A}^{E}\right)}^{2}+\left(x_{C}^{E}-x_{A}^{E}\right)\cdot {\left(y_{B}^{E}\right)}^{2}+\left(x_{A}^{E}-x_{B}^{E}\right)\cdot {\left(y_{C}^{E}\right)}^{2}\right]\\ a=\sqrt{-\frac{A}{B}}\cdot R \end{array}$$

where $\begin{array}{l} \begin{array}{cc} A= & x_{B}^{E}(x_{C}^{E}-x_{D}^{E})(x_{C}^{E}+x_{D}^{E}-x_{B}^{E})y_{A}^{E}+x_{A}^{E}(x_{D}^{E}-x_{C}^{E})(x_{D}^{E}+x_{C}^{E}-x_{A}^{E})y_{B}^{E}\\ & +(x_{B}^{E}-x_{A}^{E})(x_{A}^{E}-x_{D}^{E})(x_{B}^{E}-x_{D}^{E})y_{C}^{E}+(x_{A}^{E}-x_{B}^{E})[x_{A}^{E}x_{B}^{E}+{\left(x_{C}^{E}\right)}^{2}]y_{D}^{E} \end{array}\\ \begin{array}{cc} B= & (y_{B}^{E}-y_{C}^{E})(y_{B}^{E}-y_{D}^{E})(y_{C}^{E}-y_{D}^{E})x_{A}^{E}+(y_{C}^{E}-y_{A}^{E})(y_{C}^{E}-y_{D}^{E})(y_{A}^{E}-y_{D}^{E})x_{B}^{E}\\ & +(y_{B}^{E}-y_{A}^{E})(y_{B}^{E}-y_{D}^{E})(y_{D}^{E}-y_{A}^{E})x_{C}^{E}+(y_{C}^{E}-y_{B}^{E})(y_{A}^{E}-y_{B}^{E})(y_{A}^{E}-y_{C}^{E})x_{D}^{E} \end{array}\\ C=(x_{C}^{E}-x_{B}^{E})(y_{B}^{E}-y_{A}^{E})-(x_{B}^{E}-x_{A}^{E})(y_{C}^{E}-y_{B}^{E}). \end{array}$.

To better describe the relationship between measured diameter and measuring lasers, a constructing function *f* (*m*, *n*, *a*, *x^E^*, *y^E^*, *R*) is defined as:

(10) $$f(m,n,a,x^{E},y^{E},R)=\frac{{\left(x^{E}-m\right)}^{2}}{a^{2}}+\frac{{\left(y^{E}-n\right)}^{2}}{b^{2}}-1$$

Hence, Equation (7) can be rewritten as:

(11) $$f(m,n,a,x^{E},y^{E},R)=0$$

By successively substituting Equation (9), Equation (6), A (*x_A_*, *y_A_*), and Equation (4), Equation (11) can be rewritten as:

(12) $$f_{i}(x_{1},y_{2},x_{3},x_{4},y_{4},\theta_{1},\theta_{2},\theta_{3},\theta_{4},\gamma ,l_{1i},l_{2i},l_{3i},l_{4i},R)=0$$

where *i* represents the group number of measurements, {*l*_1i_, *l*_2i_, *l*_3i_, *l*_4i_} are the displacements of LTSs, and {*x*_1_, *y*_2_, *x*_3_, *x*_4_, *y*_4_, *θ*_1_, *θ*_2_, *θ*_3_, *θ*_4_, *γ*} are defined as the intrinsic parameters.

In conclusion, the single-module measurement model has been derived as Equation (12). In actual application, the established measurement model can be used for both calibration and measurement. In calibration procedure, *R* is the nominal value of the measured diameter, whose value has been verified by CMM. {*l*_1i_, *l*_2i_, *l*_3i_, *l*_4i_} are known variables, which can be acquired from the LTSs. By changing the relative position between measuring module and measured shaft hole, different groups of {*l*_1i_, *l*_2i_, *l*_3i_, *l*_4i_} can be obtained. The intrinsic parameters can be calculated from Equation (12), given measured diameter *R* and collections of {*l*_1i_, *l*_2i_, *l*_3i_, *l*_4i_}. In measurement process, the intrinsic parameters are inherent, whose estimated values have been calculated in calibration procedure. The objective parameter *R* can be calculated from Equation (12), by substituting the measuring values of LTSs and the estimated values of the intrinsic parameters. From the analysis above we can also see that, regardless of the relative pose between the measuring unit and measured workpiece, the inner diameter of measured shaft hole can always be calculated. As a result, the measuring unit based on LTSs is free from accurate positioning or adjustment, which will contribute to performing fast and automatic measurement.

### 4.2. Data Processing Algorithm

Estimate values of the intrinsic parameters are directly related to the final measuring accuracy. To enhance the robustness of the intrinsic parameters, the estimated values should satisfy Equation (12) while inserting different groups of measurements {*l*_1i_, *l*_2i_, *l*_3i_, *l*_4i_}. Based on this analysis, a constructive function is introduced:

(13) $$Func=\sum_{i=1}^{n}\left|f_{i}\right|$$

Our purpose is to find out the very values of the intrinsic parameters, which enable *Func* to reach the minimum value. An improved differential evolution (DE) algorithm named JADE [26] is employed here to solve this problem.

**Figure 7:**
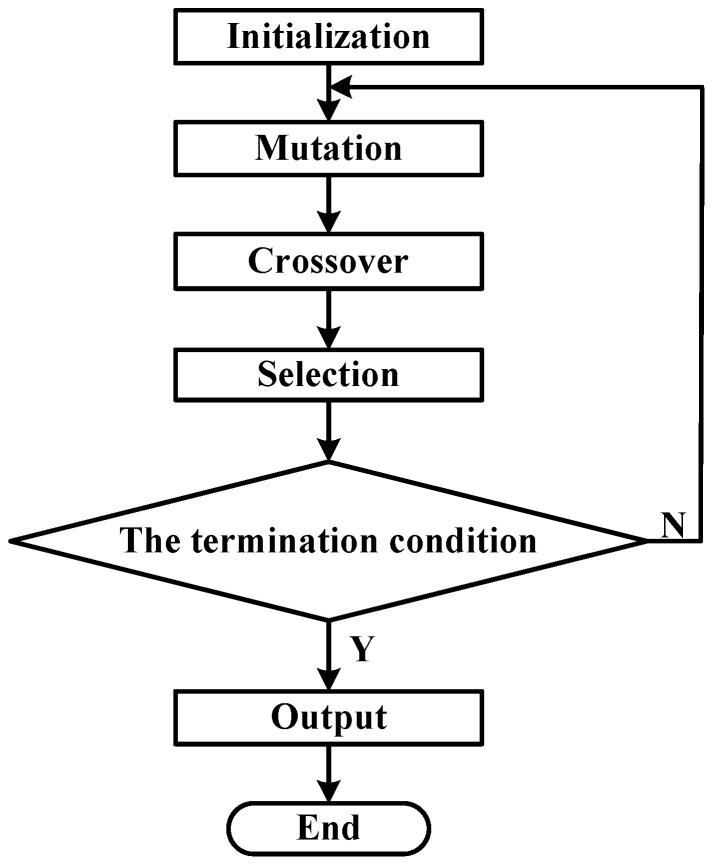
The principle of Differential Evolution algorithm.

DE algorithm, proposed by Storn and Price [25], is successfully applied to solve numerous optimization problems in diverse fields. It offers great flexibility, robustness and precision with respect to various types of functions. The performance of DE algorithm mainly depends on three control parameters (mutation control parameter *F*, crossover control parameter *Cr*, and population size *Np*) and two algorithm strategies (mutation strategy and crossover strategy). JADE improves the optimization performance of DE by implementing a new mutation strategy and updating control parameters in an adaptive manner [27]. The principle of JADE algorithm, as shown in Figure 7, can be described as follows:

(1) Initialization
  *x_i,G_* is an *D*-dimensional individual, which is defined as:
  (14) $$x_{i,G}=\left\{x_{i,G}^{1},\ldots ,x_{i,G}^{D}\right\},i=1,\ldots ,Np$$
  where *G* represents the number of generations, and *Np* is the number of individuals. The initial value of *G* is given as 0. The initial population which contains *Np D*-dimensional individuals, is generated through Equation (15):
  (15) $$x_{i,0}^{j}=x_{\min}^{j}+rand(0,1)\cdot (x_{\max}^{j}-x_{\min}^{j}),j=1,\ldots ,D.$$
  where $x_{\text{min}}^{j}$ and $x_{\text{max}}^{j}$ are, respectively, the lower and upper limits of the *j*th variable of the individual, *rand*(0, 1) is a random number, which ranges from 0 to 1, under uniform distribution. In this paper, the value of *D* is 10. {$x_{i,G}^{1}$,…,$x_{i,G}^{D}$}, respectively, correspond to the intrinsic parameters {*x*_1_, *y*_2_, *x*_3_, *x*_4_, *y*_4_, *θ*_1_, *θ*_2_, *θ*_3_, *θ*_4_, *γ*}. Considering the system design and the actual errors caused by machining and installation, the intrinsic parameters follows the constraint in Equation (16). The population size *Np* is usually defined as 10·*D*. To further improve the accuracy of parameter estimation, the population size *Np* is given as 1000 here.
  (16) $$\{\begin{array}{c} -44\leq x_{1}\leq -45\\ 44\leq y_{2}\leq 45\\ 44\leq x_{3}\leq 45\\ -0.5\leq x_{4}\leq 0.5\\ -44\leq y_{4}\leq -45\\ 0{}^{\circ}\leq \theta_{1}\leq 2{}^{\circ}\\ 0{}^{\circ}\leq \theta_{2}\leq 2{}^{\circ}\\ 0{}^{\circ}\leq \theta_{3}\leq 2{}^{\circ}\\ 0{}^{\circ}\leq \theta_{4}\leq 2{}^{\circ}\\ 0{}^{\circ}\leq \gamma \leq 2{}^{\circ} \end{array}$$
(2) Mutation
  The mutation vector *v_i,G_* is generated through Equation (17):
  (17) $$v_{i,G}=x_{i,G}+F_{i}\cdot (x_{pbest,G}-x_{i,G})+F_{i}\cdot (x_{r1,G}-x_{r2,G}),i\neq r1\neq r2.$$
  where *x_pbest,G_* represents one of the top 100·*p*% individuals in current population, *F_i_* denotes the mutation factor that varies with the make-up of population, and *r*1 and *r*2 are random numbers chosen from 1 to *Np*. The value of *p* is given as 0.1. *F_i_* is determined by the following equation:
  (18) $$\{\begin{array}{l} \begin{array}{l} F_{i,G+1}=randc_{i}(\mu_{F,G+1},0.1) \end{array}\\ \mu_{F,G+1}=(1-c)\cdot \mu_{F,G}+c\cdot mean_{L}(S_{F,G})\\ mean_{L}(S_{F,G})=\frac{\sum_{F\in S_{F,G}}F^{2}}{\sum_{F\in S_{F,G}}F} \end{array}$$
  where *F_i_* is generated in accordance with Cauchy distribution, *mean_L_*(∙) is the Lehmer mean, and *S_F_* is the set of all successful mutation factors in current population. The initial value of *μ_F_* is given as 0.5, and the value of *c* is given as 0.1 here.
(3) Crossover
  The exponential crossover operation is selected as the crossover strategy because this strategy usually has a good performance in nonlinear optimization. The trial vector $u_{i,G+1}$ is defined as:
  (19) $$u_{i,j,G+1}=\{\begin{array}{l} v_{i,j,G+1},from\;j=j_{rand}\;while\;R_{j}\leq Cr_{i}\\ x_{i,j,G},otherwise \end{array}j=1,\ldots ,D$$
  where *j_rand_* is a random number within the range [1, D], and *R_j_* is a uniform random number in the range of [0, 1]. *Cr_i_* is expressed as the equation below:
  (20) $$\{\begin{array}{l} Cr_{i,G+1}=randn_{i}(\mu_{Cr,G+1},0.1)\\ \mu_{Cr,G+1}=(1-c)\cdot \mu_{Cr,G}+c\cdot mean_{A}(S_{Cr,G}) \end{array}$$
  where *Cr_i_* is generated according to the normal distribution, *mean_A_*(∙) is the usual arithmetic mean, and *S_Cr_* is the set of all successful crossover factors in current population. The initial value of *μ_Cr_* is given as 0.5.
(4) Selection
  The offspring $u_{i,G+1}$ is defined as below:
  (21) $$u_{i}^{G+1}=\{\begin{array}{l} u_{i}^{G+1},f(u_{i}^{G+1})\leq f(u_{i}^{G})\\ x_{i}^{G},otherwise \end{array}$$
(5) Termination condition
  G = G + 1. (The generation number *G* increases by one.)
  Repeat Steps 2–5 until the value of *Func* no longer decreases in the last 100 generations.

**Figure 8:**
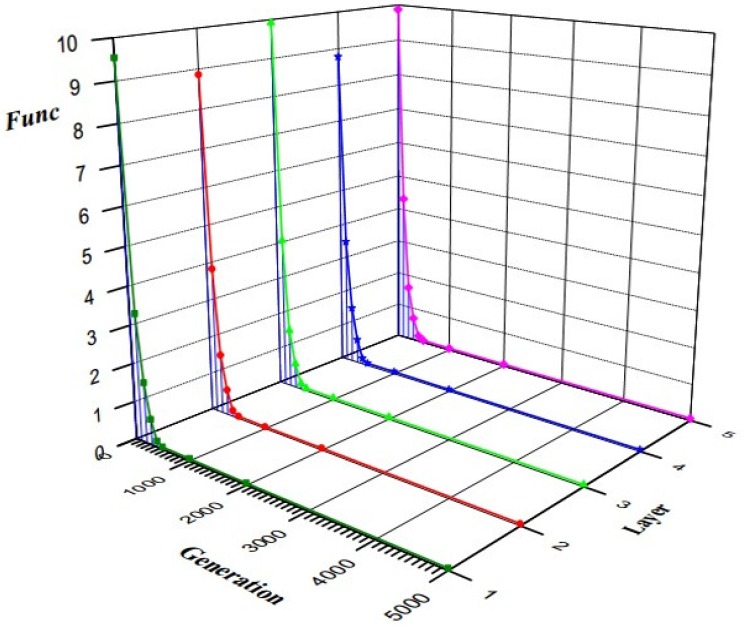
The performance of JADE for intrinsic parameter calibration.

The analysis above describes the implementation steps of JADE algorithm. The parameter setting is also given in order to facilitate other researchers to use in similar situations. In this article, 50 groups of measurements {*l*_1i_, *l*_2i_, *l*_3i_, *l*_4i_} were collected to perform parameter estimation. To improve the robustness of the intrinsic parameters, these measurements should be approximately under uniformly distributed in their measuring ranges. By substituting these groups of measurements into Equation (12), the values of the intrinsic parameters can be calculated. The performance of JADE algorithm is shown in Figure 8. The horizontal axis is the number of generations, and the vertical axis is the mean of *Func* values over 10 independent runs. From Figure 8, we can see that the values of *Func* barely decreased after 1000 generations and the program usually stopped running within 5000 generations. The optimal estimated values of the intrinsic parameters are shown in Table 1.

## 5. Experiments and Discussion

**Figure 9:**
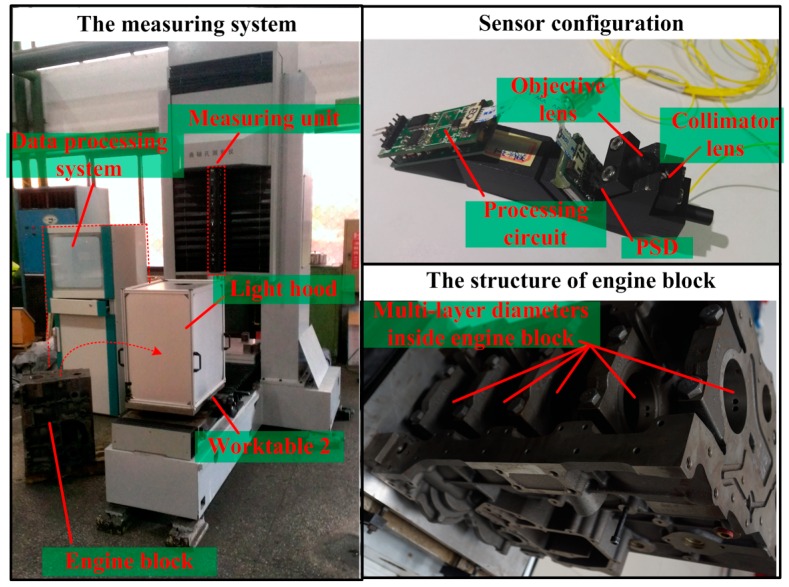
The measuring system on site.

The online measuring system for shaft holes inside engine block is shown in Figure 9. The measured workpiece is the 4 H engine block by Dongfeng Motor Co., Ltd. The light hood above the measured workpiece is to eliminate the interference caused by ambient light. The LTSs used in the measuring system are developed by ourselves. The measuring range of developed LTSs is 1.5 mm while their measuring accuracy is within 1 μm, and the stand-off distance is 0.5 mm. All the sensors’ triangulation angles are about 25°. The PSDs used in the developed LTSs are S4584-06 of Hamamatsu Photonics (Japan). The semiconductor lasers are MQW F-P LD of Rayscience Optoelectronic Innovation (China). The size of objective lens is Φ6 mm × 14 mm. Its object distance is 8.63 mm while its image distance is 9.97 mm. The software used for data processing is written in VC++2008 and runs on a computer with an Intel Core I7 3970 K 3.5 GHz and 8 GB RAM. The measurement procedure is described as follows:

(1) The measured engine block on production line is transported to proposed measuring system and finally located at Station 3.
(2) Measured engine block is transported from Station 3 to Station 2 by double-acting cylinders.
(3) The measuring unit moves down through the measured shaft holes. Data samples collected by the measuring unit are then transmitted to data processing system on RS-485 bus.
(4) The measuring results are finally computed by data processing system.

The verification tests for proposed measuring system and method were carried out using three 4 H engine blocks. The measuring data are shown in Table 2, Table 3 and Table 4. Before measurements, the standard values of measured workpieces have been verified by CMM. The level of agreement between the CMM measurement and the experiment with the new sensor can be calculated by the following equation:

(22) The measuring deviation = |Standard Value − Measuring Value|

The experimental results show that the measuring deviation is less than 4 μm and the standard deviation is less than 2 μm. In addition, the measuring time for each workpiece was nearly 3 min, including system transportation and measurement process.

**Figure 10:**
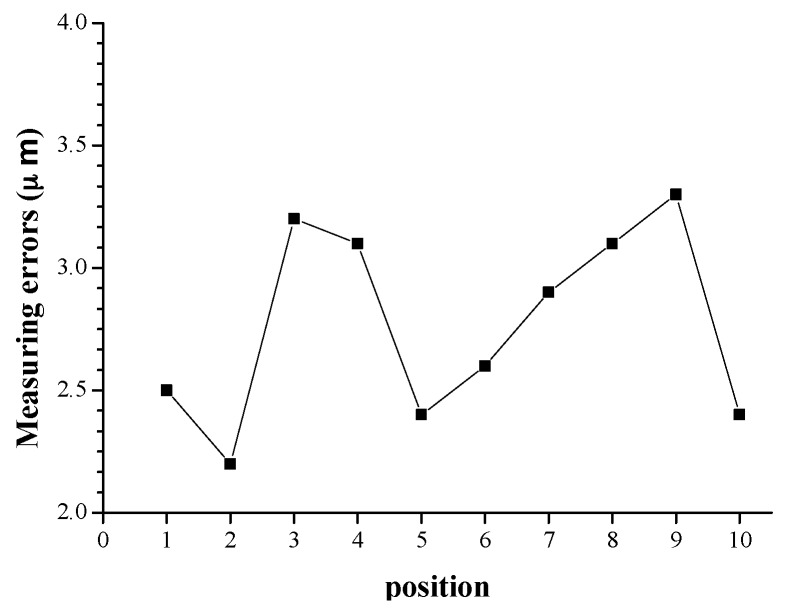
The influence of measuring position.

**Figure 11:**
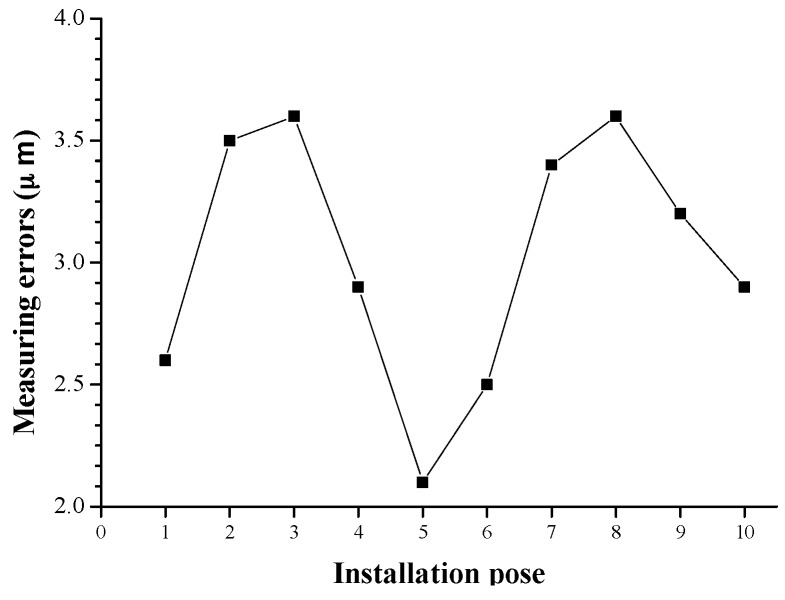
The influence of installation pose.

As analyzed in Section 4.1, the measuring unit’s installation pose and its measuring position do not impact the measuring results. This is the basis to perform fast and automatic measurement. To validate this, the engine block is firstly measured at 10 different positions, and then measured at 10 different installation poses, and the measuring results are shown in Figure 10 and Figure 11, respectively. From Figure 10 and Figure 11, it is observed that the measuring errors are all in the range from 2 μm to 4 μm, and the results coincide with the data in Table 2, Table 3 and Table 4. Consequently, it can be concluded that the measuring unit’s installation pose and measuring position do not have obvious influence on the measuring accuracy.

**Figure 12:**
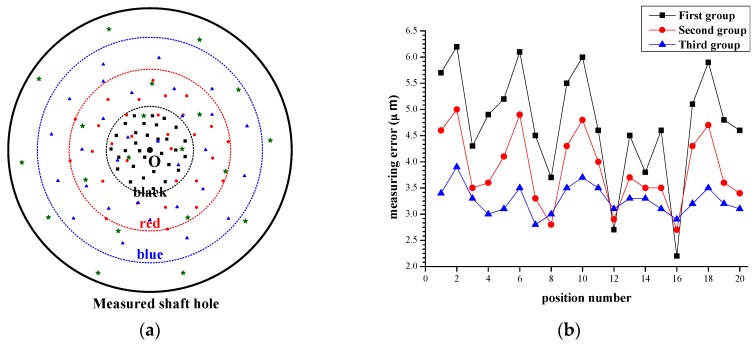
The influence of calibration data: (**a**) the position relationship between measuring module and measured shaft hole; and (**b**) the measuring errors using three groups of intrinsic parameters.

Another relevant factor that may affect the measuring accuracy is calibration data. Measuring data for calibration have vital effect on estimate values of the intrinsic parameters. Furthermore, the intrinsic parameters would affect the final measuring results. To intuitively illustrate this relationship, different data samples are employed in parameter estimation. In Figure 12a, *O* is the shaft axis of measured shaft hole. The other points in color are the shaft axis of the measuring module. Hence, the position relationship between the measured shaft hole and the measuring module can be deemed as the relative position between point *O* and the points in color. The points in black are the positions of the measuring module where we get the first sets of calibration data. The points in red are the measuring module’s positions where we acquire the second sets of calibration data. The points in blue are the positions where to obtain the third sets of calibration data. In calibration process, each set of calibration data are acquired from 30 measurements. These measurements are approximately uniformly distributed around the shaft axis of shaft hole. Three groups of intrinsic parameters can be calculated according to three sets of calibration data. To test the influence of calibration data to measuring accuracy, the engine block is measured at diverse positions using the intrinsic parameters above. The points in green are the measuring module’s positions during measurement process. The measuring errors in Figure 12b clearly illustrate that calibration data have a visible impact on the measuring accuracy. The bigger range calibration data are in (Figure 12a), the higher accuracy we can achieve (Figure 12b). Therefore, calibration data should cover their feasible ranges and better meet uniformly distributed. This conclusion is consistent with what we mentioned in Section 4.2.

## 6. Conclusions

This article presents a high-speed automatic measuring system and method for dimensional inspection of shaft holes inside engine block. The performed experiments allow validating the system and the method. The measuring results show that the measuring error is less than 4 μm and the standard deviation is less than 2 μm. The measuring time for each engine block is within 3 min. Consequently, the proposed measuring system and method provide an effective way for car engine production line to realize online quality control.

## Figures and Tables

**Table 1:** Values of the intrinsic parameters (mm, °).

Layer	The Intrinsic Parameters									
	*x*_1_	*y*_2_	*x*_3_	*x*_4_	*y*_4_	*θ*_1_	*θ*_2_	*θ*_3_	*θ*_4_	*γ*
1	44.5671	44.5723	44.5713	−0.1327	−44.6198	0.1337	0.6959	1.2318	0.5715	0.3753
2	44.7193	44.6717	44.9979	0.3336	−44.2274	1.2836	0.9078	1.1764	0.3842	0.3755
3	44.6228	44.6034	44.6315	0.2098	−44.3536	0.7832	0.7344	1.3335	1.7318	0.3751
4	44.5234	44.5171	44.5297	−0.1373	−44.5475	0.0595	0.5375	1.5518	1.6273	0.3751
5	44.6866	44.6255	44.9436	0.3091	−44.8171	0.9061	0.8224	0.5942	0.8619	0.3753

**Table 2:** Measuring data of Engine Block 1# (mm).

Layer	No. 1	No. 2	No. 3	No. 4	No. 5	Average Value	Standard Deviation	Standard Value
1	91.9972	92.0010	91.9969	91.9993	91.9966	91.9982	0.0019	91.9997
2	91.9989	91.9989	92.0002	91.9997	92.0018	91.9999	0.0012	92.0011
3	91.9972	92.0013	91.9979	91.9981	91.9970	91.9983	0.0017	91.9999
4	91.9921	91.9946	91.9928	91.9959	91.9921	91.9935	0.0017	91.9947
5	92.0002	92.0009	92.0011	92.0028	92.0035	92.0017	0.0014	92.0026

**Table 3:** Measuring data of Engine Block 2# (mm).

Layer	No. 1	No. 2	No. 3	No. 4	No. 5	Average Value	Standard Deviation	Standard Value
1	91.9931	91.9952	91.9962	91.9934	91.9956	91.9947	0.0014	91.9959
2	92.0048	92.0057	92.0061	92.0049	92.0075	92.0058	0.0011	92.0070
3	91.9975	91.9991	92.0018	91.9985	92.0006	91.9995	0.0017	91.9984
4	91.9962	91.9966	91.9976	91.9983	91.9983	91.9974	0.0010	91.9960
5	91.9993	91.9985	92.0004	91.9986	92.0012	91.9996	0.0012	91.9984

**Table 4:** Measuring data of Engine Block 3# (mm).

Layer	No. 1	No. 2	No. 3	No. 4	No. 5	Average Value	Standard Deviation	Standard Value
1	91.9956	91.9964	91.9977	91.9982	91.9956	91.9967	0.0012	91.9973
2	92.0035	92.0038	92.0047	92.0048	92.0037	92.0041	0.0006	92.0053
3	91.9958	91.9985	91.9961	91.9965	91.9971	91.9968	0.0011	91.9981
4	91.9948	91.9948	91.9972	91.9976	91.9966	91.9962	0.0013	91.9958
5	91.9978	92.0013	91.9982	91.9999	92.0008	91.9996	0.0016	92.0007
